# QuickStats

**Published:** 2015-07-10

**Authors:** FROM THE NATIONAL CENTER FOR HEALTH STATISTICS

**Figure:**
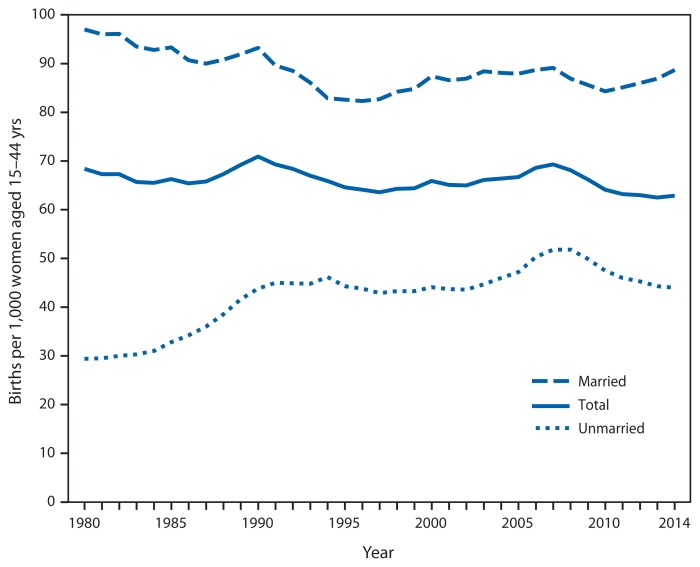
Annual Birth Rates,^*^ by Marital Status — National Vital Statistics System, United States, 1980–2014^†^ ^*^ Births per 1,000 women aged 15–44 years in each category. ^†^ Data for 2014 are preliminary.

The birth rate for married women (97.0 per 1,000) was more than three times that for unmarried women (29.4) in 1980. From1980 to the mid-1990s, the birth rate among married women generally declined, whereas the rate for unmarried women generally increased. Both rates stabilized in the mid-1990s and then increased until 2007–2008. The birth rate among unmarried women declined from 51.8 in 2007 and 2008 to 44.0 in 2014. The birth rate for married women dropped 5% during 2007–2010 but increased to 88.7 in 2014.

**Source:** Hamilton B, Martin J, Osterman M, Curtin S. Births: preliminary data for 2014. Natl Vital Stat Rep 2015;64(6). Available at http://www.cdc.gov/nchs/data/nvsr/nvsr64/nvsr64_06.pdf.

**Reported by:** Sally C. Curtin, MA, scurtin@cdc.gov, 301-458-4142.

